# Expectation confounds in psychiatric trials: insights from placebo research

**DOI:** 10.1192/j.eurpsy.2026.10319

**Published:** 2026-07-31

**Authors:** N. Huneke

**Affiliations:** University of Southampton, Southampton, United Kingdom

## Abstract

**Abstract:**

Randomized-controlled trials (RCTs) are not free of biases. For instance, expectation effects are an important confound in psychiatric RCTs. In this talk, I will explore the relationship between expectation effects, blinding integrity, and estimates of efficacy regarding active treatment and placebo. Proposed methods to mitigate these confounders and enhance the reliability of RCTs across psychiatry will be discussed. I will conclude with suggested research directions and novel approaches to reduce potential confounding by expectation effects.

**Disclosure of Interest:**

N. Huneke Grant / Research support from: I have received funding from the NIHR and MRC.

